# Broomrape infestation in carrot (*Daucus carota*): Changes in carotenoid gene expression and carotenoid accumulation in the parasitic weed *Phelipanche aegyptiaca* and its host

**DOI:** 10.1038/s41598-019-57298-7

**Published:** 2020-01-15

**Authors:** Sewar Emran, Bhagwat Nawade, Mosaab Yahyaa, Jackline Abu Nassar, Dorothea Tholl, Hanan Eizenberg, Mwafaq Ibdah

**Affiliations:** 1 Newe Ya’ar Research Center, Agricultural Research Organization (ARO), Ramat Yishay, Israel; 2 Department of Biological Sciences, Virginia Polytechnic Institute and State University, 409 Latham Hall, 220 Ag Quad Lane, Blacksburg, Virginia 24061 United States

**Keywords:** Biochemistry, Molecular biology, Plant sciences

## Abstract

Carotenogenesis has been intensively studied in carrot roots, and transcriptional regulation is thought to be the major factor in carotenoid accumulation in these organs. However, little is known about the transcriptional regulation of carotenoid biosynthetic genes concerning carotenoid accumulation during infestation by the obligate parasite *Phelipanche aegyptiaca*. HPLC analysis revealed a decrease in carotenoid levels of the different carrot cultivars when parasitized by *P*. *aegyptiaca*. Besides, we isolated and analyzed *P*. *aegyptiaca* tubercles parasitizing the various carrot root cultivars and show that they accumulate different carotenoids compared to those in non-infested carrot roots. Expression analysis of *PHYTOENE SYNTHASE* (*PSY1)* and *CAROTENOID ISOMERASE* (*CRTISO*) as well as the strigolactone apocarotenoid biosynthetic genes *DWARF27* (*D27*), *CAROTENOID CLEAVAGE DIOXYGENASE* 7 (*CCD7*) and *CCD8* revealed that their transcript levels showed significant variation in *P*. *aegyptiaca* infested carrot roots. After parasite infestation, the expression of these genes was strongly reduced, as were the carotenoid levels and this was more pronounced in the uncommon non-orange varieties. We also analyzed the parasite genes encoding D27, CCD7 and CCD8 and show that they are expressed in tubercles. This raises important questions of whether the parasite produces its carotenoids and apocarotenoids including strigolactones and whether the latter might have a role in tubercle development.

## Introduction

The parasitic weeds of the genera *Orobanche*, *Phelipanche*, and *Striga* (Orobanchaceae) are the most important agricultural weeds in many crops, particularly in carrot, tomato, sunflower, tobacco, and faba bean, causing significant crop losses in many parts of the world^1^. These obligate root parasites are completely devoid of chlorophyll and consequently dependent on their host for supply of resources, including water, nutrients, proteins, and oligonucleotides^2–5^. The parasite development is divided into pre-parasitic and parasitic stages. The pre-parasitic stage starts with seed pre-conditioning followed by germination. The parasite seed germination is induced by molecules secreted into the rhizosphere by the roots of host plants called germination stimulant^6^. Germination stimulants for root parasitic plants have been isolated from host plant root exudates, and the majority of these natural compounds are carotenoid-derived strigolactones^7,8^. The parasitic phase begins with the penetration of the parasite haustorium connecting to the host vascular tissues. The haustoria are responsible for host attachment, penetration and resource acquisition^9^. The parasite first develops a tubercle, which gives rise to a flowering spike that emerges from the soil^9–12^. The flower produces thousands of extremely small seeds, which can survive more than 15 years in a crop field until favorable environmental conditions for germination are obtained^4,9–12^.

**Figure 1:**
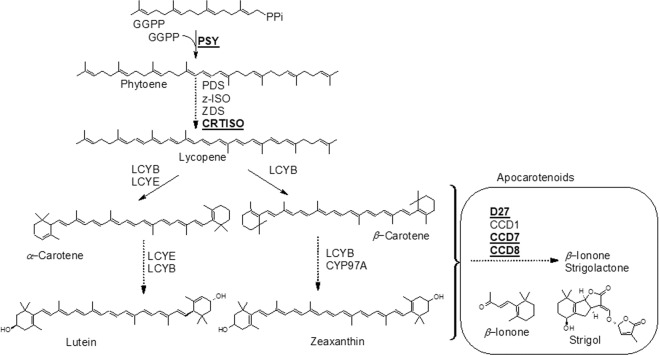
Carotenoid and apocarotenoid biosynthetic pathway in higher plants. The carrot genes investigated in this study are indicated in bold underlined style.

Carrots (*Daucus carota* L.) are popular vegetables due to their health benefits and pleasant flavor^13,14^. Terpenes are among the natural products biosynthesized during carrot root development and which directly affect root quality and flavor. They are key players constituting important pigments (carotenoids) and aroma chemicals (mono-, sesquiterpenes, and norisoprenoids)^14–18^. The accumulation of carotene compounds, such as *α*-, *β-*, *γ*-, *ζ-*carotenes, lycopene, and zeaxanthin are responsible for several types of attractive color of carrots^16,19^. Carotenoids are tetraterpenoid pigments and are synthesized de novo in the plastids of leaves, flowers, fruits, and roots, where they contribute to the red, orange and yellow colors^20^. The first step in carotenoid biosynthesis is a condensation of two molecules of geranylgeranyl pyrophosphate to produce phytoene catalyzed by phytoene synthase (PSY, EC 2.5.1.32). A series of desaturations and isomerizations converts phytoene to lycopene, including a carotenoid isomerase called CRTISO (EC 5.2.1.13) (Fig. 1). Lycopene is cyclized to yield *α*- and *β*-carotene. Subsequent oxygenation of the carotenes via additional hydroxyl, epoxy or keto groups results in the formation of xanthophylls, including lutein from *α*-carotene, and zeaxanthin, violaxanthin and neoxanthin from *β*-carotene (Fig. 1)^21–23^.

Carotenoids constitute an important precursor metabolic reservoir for the biosynthesis of bioactive compounds in plants, bacteria, fungi, and animals. Such carotenoid derivatives are formed by cleavage into apocarotenoids (norisoprenoids) through regio-specific oxidative enzymes targeting different double bonds on the carotenoid backbone^16,20,24^. In-plant cells, apocarotenoids serve as chromophores (e.g. bixin and crocin)^25^, but also as regulators of growth and development (e.g. abscisic acid)^26^. One recent popular example of the latter are strigolactones, which are particularly important in the current context, since they are (among other functions) involved in the interaction of plants with parasitic weeds as seed germination stimulants^6–8,27–30^. The strigolactones are synthesized from *β*-carotene in plastids. Their biosynthesis involves an isomerization (DWARF27, D27, EC 5.2.1.14) and two carotenoid cleavage steps catalyzed by carotenoid cleavage dioxygenase 7 (CCD7, EC 1.13.11.68) and by CCD8 (EC 1.13.11.68)^30^. Previously it has been shown that combined action of D27, CCD7, and CCD8 leads to the formation of the strigolactone precursor carlactone^6,27^. Genetic studies with mutants showed that two CCDs and P450 were responsible for the production of strigolactones^6,31,32^. Das *et al*.^33^ have previously analyzed the transcriptomes of *P*. *aegyptiaca*, *Striga hermonthica*, and *Triphysaria versicolor* and identified genes known to act in strigolactone synthesis (D27, CCD7,CCD8). In addition, Hacham *et al*.^34^ propose that *P*. *aegyptiaca* has its own metabolic mechanisms that enable the parasite to accumulate different metabolites derived from the host and/or modify/synthesize metabolites according to its own needs, which differ from those of its host. This information encouraged us to analyze the transcript accumulation of the parasite *P*. *aegyptiaca PaD27*, *PaCCD7*, and *PaCCD8* as well as carotenoid accumulations in the tubercle after carrot infestation.

Carotenoid biosynthesis and its regulation have been studied in various plant species, such as carrot, tomato, melon, tobacco, pepper, wheat, and *Arabidopsis* (reviewed by Liang *et al*.^22^. However, the impact of broomrape infestation on sugar accumulation in carrots has already been documented^35^, while the accumulation of carotenoids and the expression of carotenoid and strigolactone biosynthetic genes in *P*. *aegyptiaca* on different carrot roots have not been studied yet. In this study, the concentration and composition of carotenoids and the expression of various genes of carotenoid metabolism were investigated in the roots of five different carrot cultivars infested or non-infested by *P*. *aegyptiaca* and in the tubercles of the parasitic plant. We show here that the concentration of carotenoids and the expression of (apo)carotenoid biosynthetic genes from different carrot cultivars were dramatically reduced in the *Phelipanche*-infested carrot root. Surprisingly, alterations in these parameters not only concern host roots but also the tubercles of the parasite.

## Results

### Changes in the concentration of carotenoids in *Phelipanche*-infested and non-infested carrot roots cultivars

**Figure 2:**
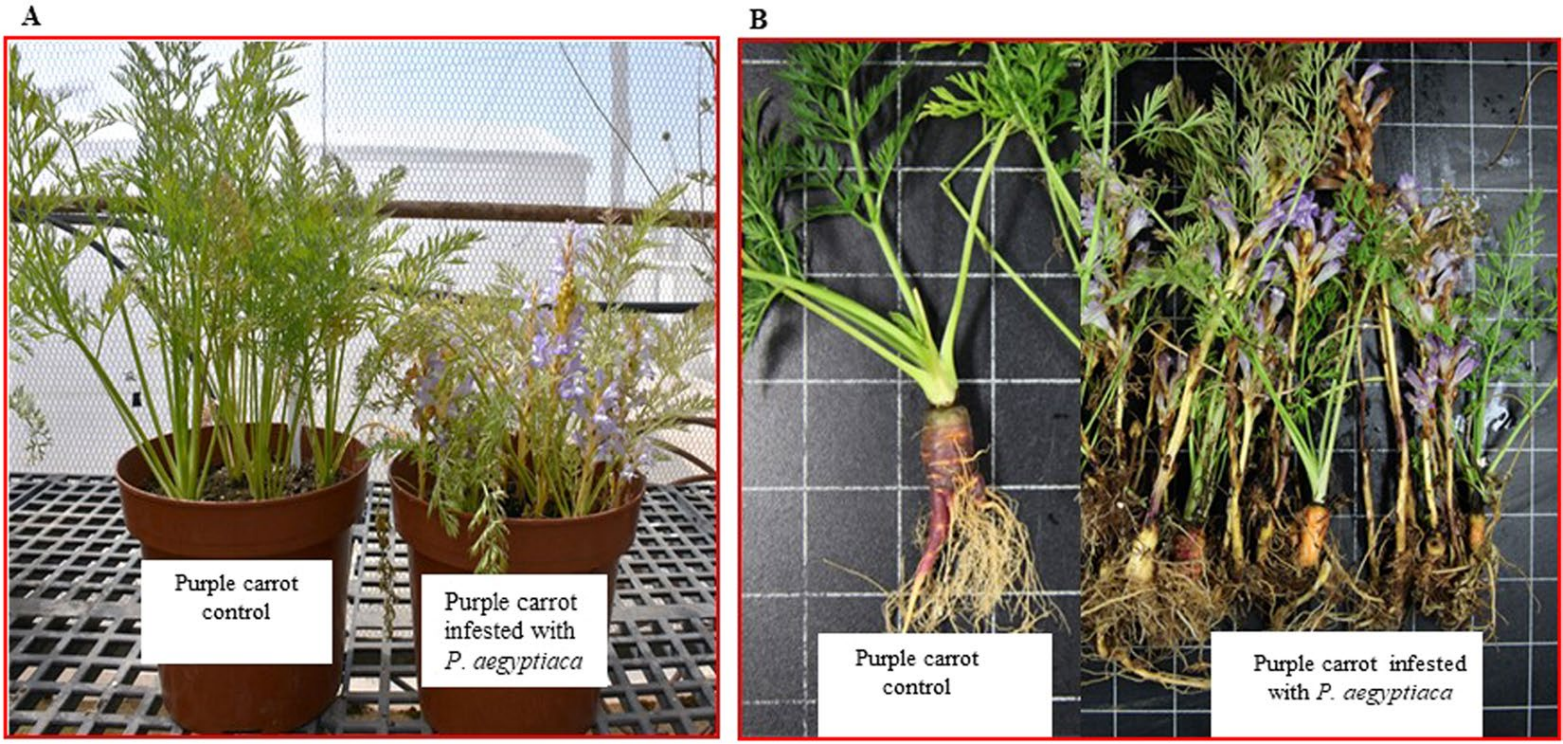
Photograph of purple carrot root without or with the obligate parasite *Phelipanche aegyptiaca* infestation as representative of all carrot cultivars. (**A**) Flowering *P*. *aegyptiaca* attached to the purple carrot variety (right image) compared to the non-infested control plant (left image). (**B**) Shoots and roots of *P*. *aegyptiaca* attached to the purple carrot root (right image) compared to non-infested control (on the left).

**Figure 3:**
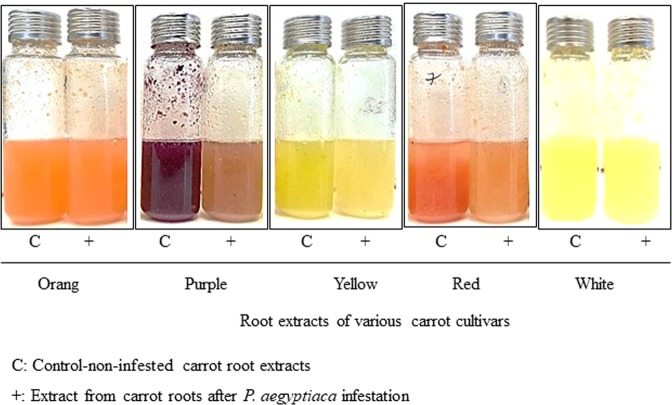
Comparison of solution colors of the various carrot root extracts with or without obligate parasite *Phelipanche aegyptiaca* infestation. C: Control non-infested carrot root extracts; +: Extract from carrot roots after *P*. *aegyptiaca* infestation.

The impact of *P*. *aegyptiaca* on the development of carrots cultivars (orange, purple, red, yellow and white), observed after 12 weeks of parasitism and results showed the drastic effect on all carrot cultivars (Fig. 2). The growth of *P*. *aegyptiaca* infected plants has been stunted in all the cultivars. The leaves of infested carrot were much less developed than those of the control, non-parasitized plants (Fig. 2A). *P*. *aegyptiaca* prevented the formation of the normal shape and size of carrot roots (Fig. 2B). Non-infested carrot roots of the different color cultivars developed a large storage root, which is conical-shaped and highly pigmented. In contrast, the *Phelipanche*-infested carrot roots were very small, abnormally shaped and less pigmented (Figs. 2 and 3).

**Figure 4:**
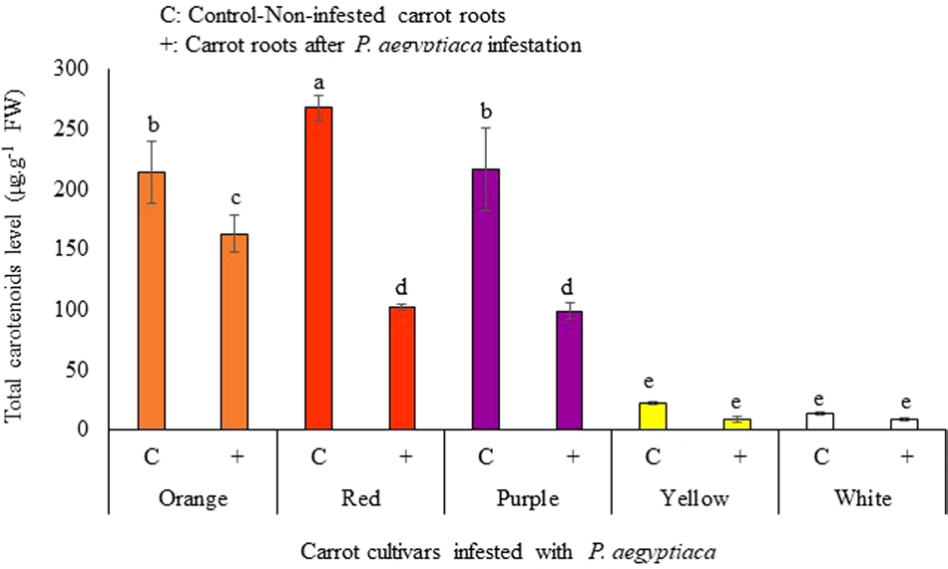
HPLC analysis of total carotenoid levels in different carrot cultivars. Carotenoid standards were identified on the basis of commercial standards. All analyses were performed using five biological replicates. C: Control non-infested carrot roots; +: Carrot roots after *P*. *aegyptiaca* infestation. Means with the same letter are not significantly different from each other (Tukey’s test, *P* ≤ 0.05).

The significant differences were observed for total carotenoids levels in infected and non-infected carrot cultivars (Fig. 4). The typical orange carrot contains large amounts of *α*- and *β*-carotene, the red carrot contains high lycopene, the color of the yellow carrot is due to lutein, and the purple carrot contains high levels of anthocyanin besides *α*- and *β*-carotene. All five carrot cultivars showed decreased accumulation in total carotenoid content in the *Phelipanche*-infested root, as compared to the non-infested carrot roots (Fig. 4). In case of non-infested carrots,the highest accumulation of total carotenoids was recorded in red carrot (276 µg.g^−1^), followed by purple (216 µg.g^−1^), orange carrots (214 µg.g^−1^), yellow (21 µg.g^−1^) and white carrots (13 µg.g^−1^) (Fig. 4). In contrast, *Phelipanche*-infested carrots showed drastic reduction in total carotenoids as compared to the non-infested roots. Among the carrot cultivars, red, yellow and purple carrot roots showed the highest reduction percentage in the accumulation of total carotenoids as compared to the non-infested carrot roots, while the *Phelipanche*-infested orange, and white carrot showed less decrease (Fig. 4).

**Figure 5:**
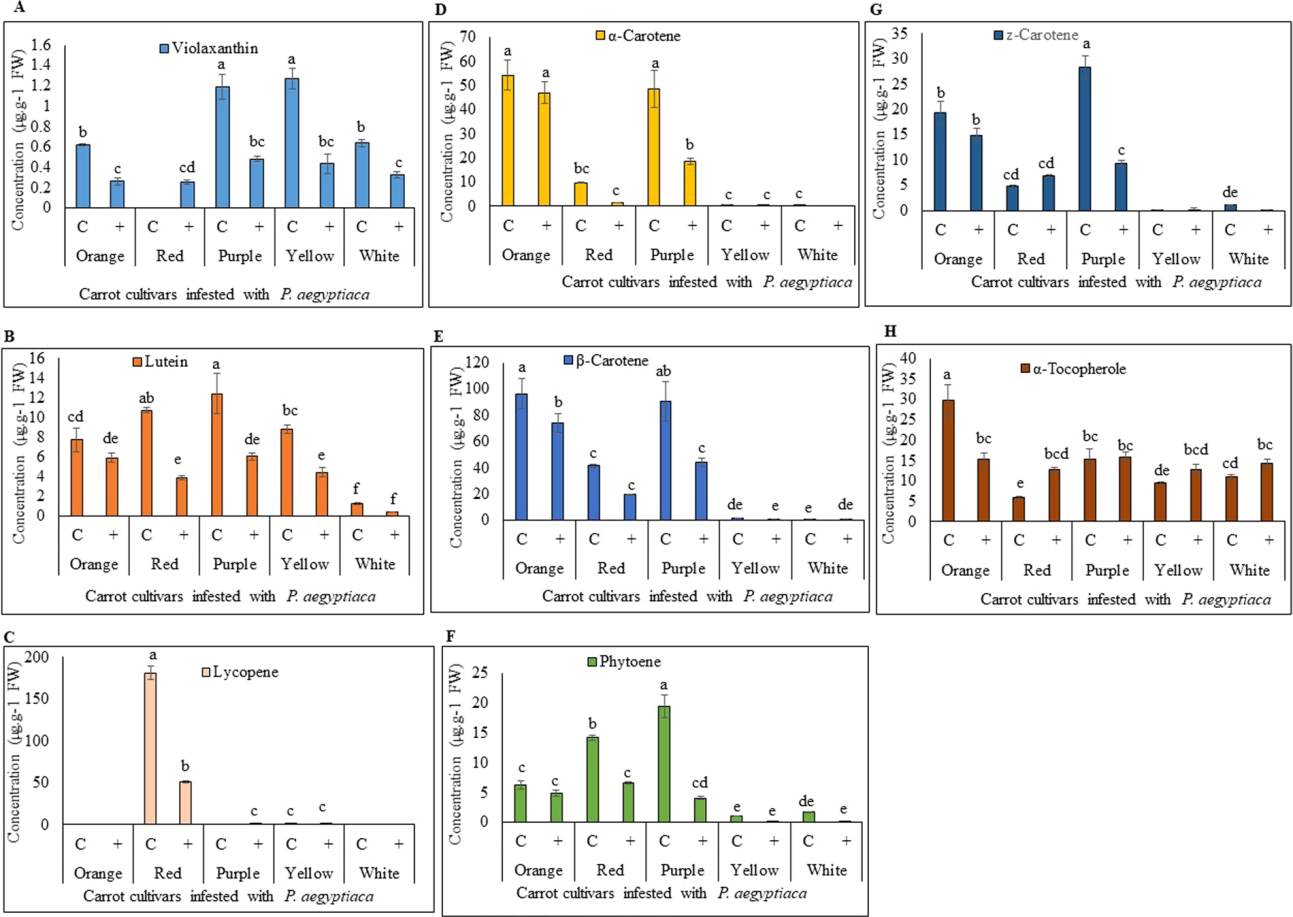
HPLC analysis of individual carotenoids accumulation in different carrot cultivars infested with *P*. *aegyptiaca*. (**A**) Violaxanthin, (**B**) Lutein, (**C**) Lycopene, (**D**) *α*-Carotene, (**E**) *β*-Carotene, (**F**) Phytoene, (**G**) *z*-Carotene, (**H**) *α* -Tocopherol. Carotenoid standards were identified on the basis of commercial standards. All analyses were performed using five biological replicates. C: Control non-infested carrot roots; +: Carrot roots after *P*. *aegyptiaca* infestation. Means with the same letter are not significantly different from each other (Tukey’s test, *P* ≤ 0.05).

There was also a significant difference between the various carrot cultivars in terms of changes in the accumulation of individual carotenoids upon infestation by *P*. *aegyptiaca*. For example, the *Phelipanche*-infested orange carrot showed a reduction of 12% in *α*-carotene and 22% in *β*-carotene, the red carrot manifested a reduction of 84% in *α*-carotene and 53% in *β*-carotene, and the purple carrot showed a reduction of 62% in *α*-carotene and 50% in *β*-carotene after the parasitic attachment (Fig. 5). The *Phelipanche*-infested roots of the yellow and the white carrot cultivars showed a lutein reduction of 59% and 33%, respectively (Fig. 5).

**Figure 6:**
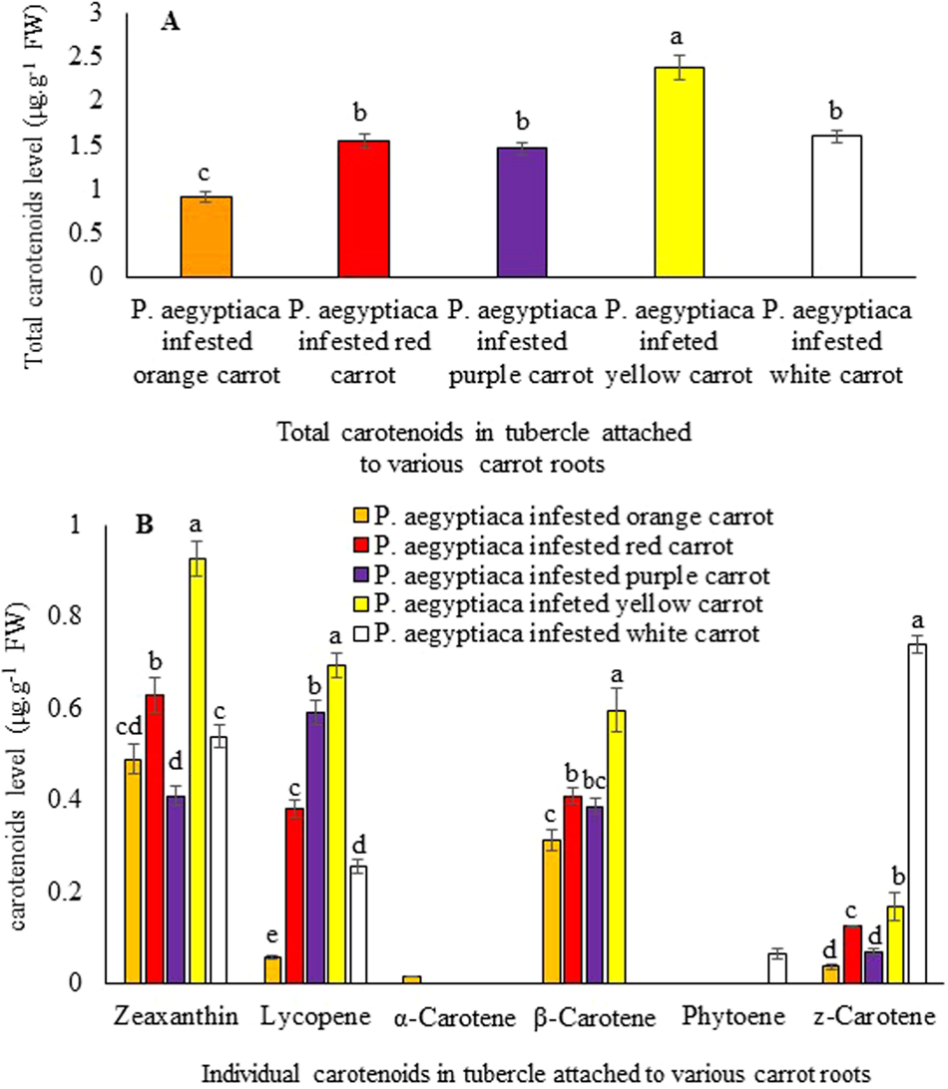
HPLC analysis of (**A**) total carotenoids, (**B**) individual carotenoids in the tubercles of the parasitic organs of *P*. *aegyptiaca* after infestation. Carotenoid standards were identified on the basis of commercial standards. All analyses were performed using five biological replicates.

Further, we also analyzed the *P*. *aegyptiaca* tubercles from each host cultivar for total carotenoids and individual carotenoid levels. Interestingly, we were able to show for the first time that the *P*. *aegyptiaca* tubercle accumulates various carotenoids in µg levels (Fig. 6). The highest level of total carotenoids was found in *P*. *aegyptiaca* tubercles attached to the yellow carrot exhibiting 2.5 µg.g^−1^ fresh weight (FW) (Fig. 6A). There was a significant difference in carotenoid accumulation between the *Phelipanche* tubercles attached to different carrot cultivars. For example, the tubercles attached to the yellow carrot had the highest level of zeaxanthin, lycopene, and *β*-carotene, as compared to the carotenoid levels of *P*. *aegyptiaca* tubercle attached to the other carrots (Fig. 6B). *α*-Carotene was found only in the *P*. *aegyptiaca* tubercle attached to the orange carrot, phytoene was found only in *P*. *aegyptiaca* tubercle attached to the white carrot, and *P*. *aegyptiaca* tubercles attached to the white carrot accumulated the highest levels of *z*-carotene (Fig. 6B).

### Analysis of transcript abundance

The transcript levels of carrot *DcPSY1*and *DcCRTISO* as well as *D27*, *CCD7*, and *CCD8* from both carrot and *P*. *aegyptiaca*, were analyzed by qRT-PCR in the same samples that were also used in the HPLC analysis; this was done in order to find out whether the changes in carotenoid accumulation and composition due to infestation of the different carrot cultivars by the parasite could be related to changes in the expression of carotenoid biosynthetic genes.

**Figure 7:**
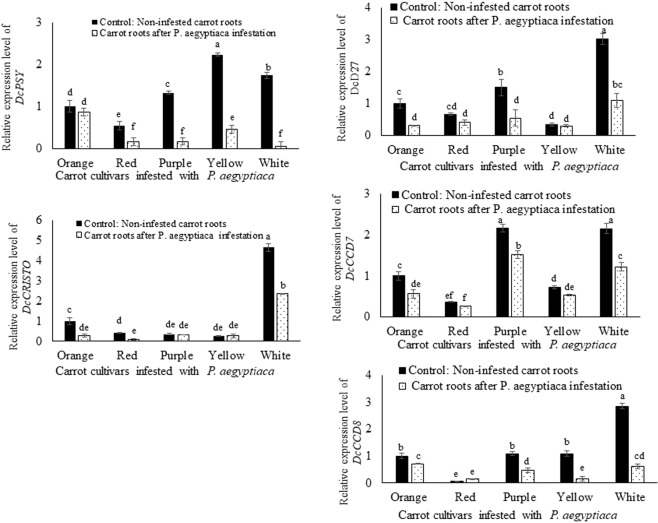
qRT-PCR determination of transcript levels of carrots (*D*. *carota*) *DcPSY1*, *DcCRTISO*, *DcD27*, *DcCCD7*, and *DcCCD8* in roots of different carrot cultivars before and after the infestion by obligate parasite *P*. *aegyptiaca*. Quantification of *DcPSY1*, *DcCRITISO*, *DcD27*, *DcCCD7* and Dc*CCD8* transcript levels by real-time RT-PCR analysis normalized to equal levels of actin transcripts. All analyses were performed using three biological replicates. C: Control non-infested carrot roots; +: Carrot roots after *P*. *aegyptiaca* infestation. Means with the same letter are not significantly different from each other (Tukey’s test, *P* ≤ 0.05).

Transcripts of *DcPSY1*, *DcCRTISO*, *DcD27*, *DcCCD7*, and *DcCCD8* were detected in all carrot root samples, showing that all the investigated genes were expressed differently in the control carrot roots and in the infested roots (Fig. 7). All of the genes showed decreased transcript levels in the infested carrot roots (Fig. 7), which is consistent with the accumulation of reduced levels of total carotenoids in the carrot roots (Fig. 4). The non-infested white carrot showed highest level expression for genes *DcCRTISO DcD27* and *DcCCD8* while *DcPSY1*, and *DcCCD7* showed highest expression in yellow and purple carrots respectively (Fig. 7). *P*. *aegyptiaca* infestation resulted in reduction of expression of *DcD27*, *DcCCD8* and *DcCCD8* genes by 2.7 fold, 1.8 fold, and 4.5 fold respectively in white carrots while yellow carrots showed 1.1, 1.3, 6.5 fold reduction in expression of these genes after *P*. *aegyptiaca* infestation. The highest reduction was observed for *DcPSY1* gene for white carrot which displayed 37 fold reduction in *P*. *aegyptiaca* infested roots as its counterpart non-infested root (Fig. 7).

**Figure 8:**
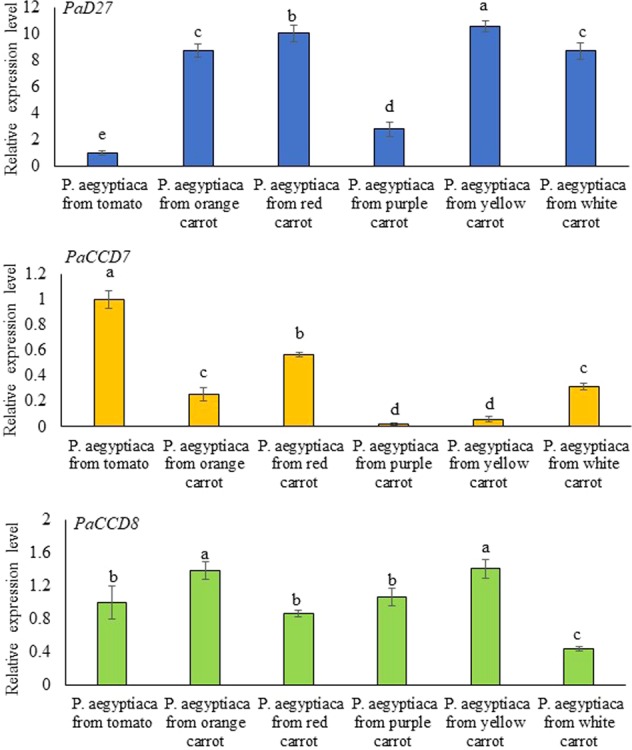
qRT-PCR determination of transcript levels of *P*. *aegyptiaca PaD27*, *PaCCD7* and *PaCCD8* in the tubercles of the parasitic organs after infested carrot and tomato (as a control) roots. Quantification of *PaD27*, *PaCCD7* and *PaCCD8* transcript level by real-time RT-PCR analysis normalized to equal levels of actin transcripts. All analyses were performed using three biological replicates. Means with the same letter are not significantly different from each other (Tukey’s test, *P* ≤ 0.05).

As for the parasite genes, only transcripts of *PaD27*, *PaCCD7*, and *PaCCD8* were targeted and detected in tubercle tissue. Actin is used as a reference gene and compared with the expression of *P*. *aegyptiaca PaD27*, *PaCCD7* and *PaCCD8* in the tubercles of the parasitic organs after infested tomato roots (as a control), previously demonstrated to be involved in the strigolactone biosynthetic pathway^29^ (Fig. 8). To make sure that we detect the parasite genes here, we blasted the primers against carrot gene/transcriptome databases and showed that the sequences in the regions targeted by the primers in the parasite were different from those in the carrot *DcD27*, *DcCCD7* and *DcCCD8* genes (Supplementary Fig. S1). There was a significant difference in expression level of *PaD27*, *PaCCD*7 and *PaCCD8* between the *P*. *aegyptiaca* tubercles attached to different carrot cultivars (Fig. 8). For example, *PaD27* showed the highest level of expression in all *P*. *aegyptiaca* tubercles attached to carrot roots, as compared to the expression of *PaCCD7* and *PaCCD8* (Fig. 8). *PaCCD7* showed the highest expression in tubercles attached to red carrot followed by white carrot tubercles and lowest expression was found in tubercles from purple carrot In case of *PaCCD8* the expression pattern recorded in decreasing order i.e. tubercles from yellow showed highest expression followed by orange, purple, red and white carrots (Fig. 8). The *P*. *aegyptiaca PSY1* and *CRTISO* could not be analyzed by qRT-PCR due to the absence of the respective sequences in the parasitic plant genome database (http://ppgp.huck.psu.edu/).

## Discussion

In this study, HPLC analysis of carotenoids and quantitative RT-PCR were used to compare the content of total and individual carotenoids with the expression of five carotenoid biosynthetic genes after broomrape infestation in orange, purple, yellow, white, and red carrot cultivars, as well as in the *P*. *aegyptiaca* tubercles.

The growth and development of the various infested carrots were reduced in comparison to non-infested plants, as indicated by root size, shape and pigment profiles. The reduced size and abnormal shape of the infested roots of carrots of various colors were associated with a decrease in pigmentation, i.e. in carotenoid accumulation in the roots (Figs. 2–5). The stunted growth of crop plants that were parasitized by broomrapes was reported for different host-parasite associations and under different growth conditions^10,35–38^. Westwood^39^ discussed the alteration in the physiology/metabolism of the host, and/or the reduction in the potential of the infested host root system to take up nutrients/water from the soil leading to a reduction in assimilate production. Schaffer *et al*.^35^ showed that the soluble sugar content of mature carrot roots grown in broomrape-infested fields was dramatically reduced. Changes both in protein and free amino acid pools of the broomrape-infested carrot roots were also reported^40^. Moreover, changes in morphological traits do not affects carotenoid accumulation in carrot roots^41^. There was no significant effect of root size on the various carotenoid pigments even though the contents were decreased with increasing root size^42^. Similarly, carrots heavily infested with broomrape showed lower sugar content even when the plants have healthy looking roots^35^.

In the current study, we have described the reduction in the total host carotenoids, of about 24% in orange infested carrot roots, 61% in red, 54% in purple, 60% in yellow, and 38% in white infested carrot roots, compared to non-infested roots (Fig. 4). Unexpectedly, we found carotenoid accumulation in the tubercles of *P*. *aegyptiaca* (Fig. 6). The *P*. *aegyptiaca* tubercles parasitizing the white and yellow carrot cultivars produced the highest amounts of total carotenoids than tubercles that grew on other cultivars (Fig. 6). Although the orange, purple and red cultivars had significantly larger amounts of total carotenoids than the white did and the yellow carrots did, yet this difference was not reflected in their respective parasite tubercles. In addition, our analysis revealed that *P*. *aegyptiaca* tubercles accumulated different carotenoids, as compared to those found in the carrot root itself. For example, zeaxanthin was found to accumulate only in the *P*. *aegyptiaca* tubercles while violaxanthin and lutein were found to accumulate only in carrot cultivars but not in *P*. *aegyptiaca* tubercles (Figs. 5 and 6). Similarly, different studies reported the differential accumulation of metabolites in the parasitic plant from their hosts including free amino acids^43^, mannitol^44^, total soluble protein and phenolic compounds^34^, amino acids^45,46^, starch^47,48^. The parasite accumulates different metabolites when parasitizing different hosts, further support the assumption that the *P*. *aegyptiaca* may alter carotenoids obtained from the host and utilized these imported carotenoids for the synthesis of additional carotenoid compounds. Thus indicates the self-regulating metabolism in parasitic plants and their ability to alter, or reduce metabolites derived from the host.

On the basis of these findings we propose that regulation of expression of endogenous carotenoid biosynthetic genes in *Phelipanche* might change following certain, yet unknown, cues of its host or following an internal developmental program. However, evidence of this possibility is still missing, and *P*. *aegyptiaca* may have imported the carotenoids from the host. It has already been shown that the parasite can exchange certain molecules and macromolecules with host plants^9,39^. Translocation of molecules and macromolecules from the hosts to the parasitic plants have been well documented^49^. Molecular translation between host and parasite ranges from the movement of radiolabel sugar^50^, silencing-signal siRNA^38,51^, herbicides^52^, mRNA transcripts^53^ and plant viruses^54^ to the movement of protein^55^.

The expression profiles presented here of carrots *DcPSY1*, *DcCRTISO*, *DcD27*, *DcCCD7*, and *DcCCD8* genes were dramatically altered following an attack by the parasitic plant *P*. *aegyptiaca*, and the (apo)carotenogenic transcript levels decreased in the various carrot roots for all examined genes after the parasitic attachment of *P*. *aegyptiaca* (Fig. 7). This might indicate that the differential transcriptional regulation of these genes was the cause of the reduced pigmentation of the carrot cultivars (Figs. 2 and 3). We also assumed that the reduction in attachment stimulation activity was due to reduced production of strigolactones, although quantitative and/or qualitative changes in the strigolactone level in the root exudates were not measured in the current study.

Furthermore, the expression profile of *P*. *aegyptiaca PaD27*, *PaCCD7*, and *PaCCD8* genes suggest that *P*. *aegyptiaca* not only retained but also expresses functional genes involved in strigolactone biosynthesis (Fig. 8), resembling those in the host plant. Similarly, putative strigolactone biosynthesis genes were expressed in *Striga* (*ShCCD7*, *ShCCD8*) and the expression patterns of these genes were different from those of host *AtCCD7* and *AtCCD8* in *Arabidopsis*^56^. Thus, it is conceivable that the parasite produces its own strigolactones not to boost seed germination but rather may regulate shoot branching patterns in parasites just as in other eudicots. Previously, Das *et al*.^33^ reported the identification of several strigolactones (apo)carotenoid biosynthetic genes from *S*. *hermontica* and *P*. *aegyptiaca* and revealed that these genes are all present with apparently full length-coding sequences. It has been shown that in plants strigolactones are secreted by epidermal cells to the rhizosphere but it is also formed from the root parenchyma and phloem, where carotenoids accumulate, and from where strigolactones are transported to other organs and act as a plant hormone. They thus also act as important internal mediators of host plant development including root and shoot architecture^30^. De novo assembly and characterization of the transcriptome of parasites of Orobanchaceae have uncovered genes associated with strigolactone biosynthesis. Here we present the functional expression of these genes in *P*. *aegyptiaca*. The direct measurement of strigolactones in different parasites tissues was not successful due to the extremely low concentration of putatively present strigolactones was below the detection limit^33,56^. Any potential role of strigolactones in tubercle development may, therefore, be independent of the seed germination induction function already known and rather be compared to the developmental roles of strigolactones in host plants. There is a possibility that the endogenous strigolactones do have a role in germination but are not sufficient to induce germination. Unfortunately, we could not determine the expression of carotenoid biosynthetic genes of the parasite and whether the carotenoids identified in the tubercles are produced by the parasite or are somehow obtained from the host root for further processing by parasite enzymes.

Since *P*. *aegyptiaca* tubercles parasitizing the various carrot root cultivars accumulated different carotenoids compared to those in the carrot roots themselves, this could argue for an independent synthesis by the parasite.

In summary, next to the negative effects of infestation on host carotenoid biosynthesis our data has raised new questions about whether and how carotenoids and potentially also apocarotenoids such as strigolactones are biosynthesized from parasite enzymes in the *P*. *aegyptiaca* tubercles.

## Methods

### Chemicals

HPLC-grade acetonitrile, methanol, dichloromethane, hexane, acetone, ethanol, triethylamine, dichloromethane (Ch_2_Cl_2_), methyl *tert*-butyl ether (MTBE), lycopene, lutein, *α*-and, *β-*carotenes were purchased from Sigma-Aldrich.

### Plant material

Carrot (*Daucus carota* subsp. *sativus*) cultivars of various colors, orange, purple, yellow, and white, were grown at the “Newe Yaar” Research Center in northern Israel, under standard field irrigation and fertilization conditions. Five carrot plants for each cultivar were allowed to grow in 2 L pots that were infested or non-infested with seeds (50 ppm) of the parasitic weed *P*. *aegyptiaca* for about 12 weeks. The freshly harvested 12 weeks old carrot roots and the freshly harvested parasitic tubercles, which were attached to the carrot roots, were crushed separately in liquid nitrogen and stored at −80 °C for carotenoid and transcript analysis.

### Carotenoid content and composition

Carotenoids pigments were extracted from fresh carrot roots and *P*. *aegyptiaca* tubercles (1 g). The samples were extracted in hexane/acetone/EtOH (2/1/1, v/v/v) and saponification was performed in 8% (w/v) KOH for 5 min. The saponified material was extracted twice with hexane, which was then evaporated in vacuo. The solid pellet was re-suspended in 400 µl of MeCN/MeOH/CH_2_Cl_2_ (45/5/50, v/v/v) and passed through a 0.2-µm nylon filter before HPLC analyses^16^. Samples for carotenoid extraction were taken from three carrot roots of each of the cultivar, and from tubercles of the parasitic organs.

### HPLC analyses

Quantification of carotenoids was done using HPLC (Waters, Milford, MA, USA) equipped with a PDA detector (Waters 996), a column (250 × 4.6 mm i.d.; 4 mm), and a Nova-Pak Sentry Guard cartridge according to Ibdah *et al*.^57^, and Yahyaa *et al*.^16^. The filtered extracts (40 microliters) were injected into a 2996 Waters HPLC and the flow rate was kept at 1.5 mL.min^−1^ at 30 °C, the mobile phase consisted of solvent acetonitrile/methanol/dichloromethane (75/20/5, v/v/v) containing 0.05% (v/v) triethylamine. Detection occurred between 260 and 600 nm. The data were analyzed using the MILLENIUM software. All analyses were performed using five biological replicates.

### Transcript analysis

Total RNA (5 µg) was extracted from carrot cultivars and from the *P*. *aegyptiaca* tubercles by using the Spectrum Plant Total RNA Kit (Sigma-Aldrich). The RNA was reverse-transcribed using an oligo primer and the SuperScript II first-strand system (Invitrogen) for real-time RT-PCR analysis of carotenoid biosynthetic genes, e.g. *DcPSY1 DcCRTISO*, *DcD27*, *DcCCD7*, *DcCCD8*, *PaD27*, *PaCCD7*, and *PaCCD8*.

qRT-PCR was performed on an Applied Biosystem Step One Plus Real-Time PCR System (Life Technology) using Absolute Blue qPCR SYBR Green ROX Mix (Tamar Laboratory Supplies LTD, Israel), using 5 ng reverse-translated total RNA and 100 ng primers (Supplementary Table S1). Relative quantification of gene expression was performed using as reference the housekeeping gene actin from *D*. *carota* and from *P*. *aegyptiaca* with primers described in Supplementary Table S1. The difference in relative expression levels of all targeted gene*s* were calculated from ^2−^ΔΔCt value after normalization of data to actin. All analyses were performed using three biological replicates.

### Statistical analyses

Data for all experiments are expressed as the mean ± standard error (SE) for five biological repeats. One-way analysis of variance was used to evaluate the experimental data and Tukey’s test was used to detect significant differences (*P* ≤ 0.05) between the mean values. All statistical analyses were performed using JMP software (version, SAS Institute 2013).

## Supplementary information

Supplementary Table

Supplementary Information

## References

[1] Parker C (2009). Observations on the current status of Orobanche and Striga problems worldwide. Pest Manag. Sci..

[2] Aly R (2013). Trafficking of molecules between parasitic plants and their hosts. Weed Res..

[3] Aly R (2011). Movement of protein and macromolecules between host plants and the parasitic weed Phelipanche aegyptiaca Pers. Plant Cell Reports.

[4] Irving LJ, Cameron DD (2009). You are what you eat: interactions between root parasitic plants and their hosts. Adv. Bot. Res..

[5] Westwood JH, Roney JK, Khatibi PA, Stromberg VK (2009). RNA translocation between parasitic plants and their hosts. Pest Manag. Sci..

[6] Yoneyama, K., Ruyter-Spira, C. & Bouwmeester, H. In *Parasitic Orobanchaceae* (eds Joel, D. M., Gressel, J. & Musselman, L. J.) Ch. 10, 167–194 (Springer, 2013).

[7] Xie X, Yoneyama K, Yoneyama K (2010). The strigolactone story. Annu. Rev. Phytopathol..

[8] Yoneyama K, Awad AA, Xie X, Yoneyama K, Takeuchi Y (2010). Strigolactones as germination stimulants for root parasitic plants. Plant Cell Physiol..

[9] Joel, D. M. In *Parasitic Orobanchaceae* (eds Joel, D. M., Gressel, J. & Musselman, L. J.) Ch. 3, 25–60 (Springer, 2013).

[10] Aly R, Dubey NK, Yahyaa M, Abu-Nassar J, Ibdah M (2014). Gene silencing of CCD7 and CCD8 in *Phelipanche aegyptiaca* by tobacco rattle virus system retarded the parasite development on the host. Plant Signal Behav..

[11] Joel, D. M. In *Parasitic Orobanchaceae* (eds Joel, D. M., Gressel, J. & Musselman, L. J.) Ch. 2, 21–23 (Springer, 2013).

[12] Pérez-de-Luque, A. In *Parasitic Orobanchaceae* (eds Joel, D. M., Gressel, J. & Musselman, L. J.) Ch. 5, 75–86 (Springer, 2013).

[13] Block G (1994). Nutrient sources of provitamin a carotenoids in the American diet. Am. J. Epidemiol..

[14] Surles RL, Weng N, Simon PW, Tanumihardjo SA (2004). Carotenoid profiles and consumer sensory evaluation of specialty carrots (*Daucus carota*, L.) of various colors. J. Agric. Food Chem..

[15] Kreutzmann S, Thybo AK, Edelenbos M, Christensen LP (2008). The role of volatile compounds on aroma and flavour perception in coloured raw carrot genotypes. Int. J. Food Sci. Technol..

[16] Yahyaa M (2013). Formation of norisoprenoid flavor compounds in carrot (*Daucus carota* L.) roots: characterization of a cyclic-specific carotenoid cleavage dioxygenase 1 gene. J. Agric. Food Chem..

[17] Yahyaa M (2015). Identification and characterization of terpene synthases potentially involved in the formation of volatile terpenes in carrot (*Daucus carota* L.) roots. J. Agric. Food Chem..

[18] Ibdah, M., Muchlinski, A., Yahyaa, M., Nawade, B. & Tholl, D. In *The Carrot Genome* (eds Simon, P., Iorizzo, M., Grzebelus, D. & Baranski, R.) Ch. 16, 279–293 (Springer, Cham, 2019).

[19] Simon PW, Wolff XY (1987). Carotenes in typical and dark orange carrots. J. Agric. Food Chem..

[20] Walter MH, Strack D (2011). Carotenoids and their cleavage products: biosynthesis and functions. Nat. Prod. Rep..

[21] Hirschberg J (2001). Carotenoid biosynthesis in flowering plants. Curr. Opin. Plant Biol..

[22] Liang, M.-H., Zhu, J. & Jiang, J.-G. Carotenoids biosynthesis and cleavage related genes from bacteria to plants. *Crc*. *Cr*. *Rev*. *Food Sci*., 1–20 (2017). 10.1080/10408398.2017.1322552 28609133

[23] Young, A. J. & Pallett, K. E. In *Antioxidants in higher plants* (eds Alscher, R. G. & Hess, J. L.) Ch. 3, 59–89 (CRC Press, 2017).

[24] Nawade B (2020). Analysis of apocarotenoid volatiles during the development of *Ficus carica* fruits and characterization of carotenoid cleavage dioxygenase genes. Plant Sci..

[25] Bouvier F, Suire C, Mutterer J, Camara B (2003). Oxidative remodeling of chromoplast carotenoids: identification of the carotenoid dioxygenase *CsCCD* and *CsZCD* genes involved in Crocus secondary metabolite biogenesis. Plant Cell.

[26] Zeevaart J, Creelman R (1988). Metabolism and physiology of abscisic acid. Annu. Rev. Plant Physiol. Plant Mol. Biol..

[27] Alder A (2012). The path from *β*-carotene to carlactone, a strigolactone-like plant hormone. Science.

[28] Bouwmeester HJ, Roux C, Lopez-Raez JA, Becard G (2007). Rhizosphere communication of plants, parasitic plants and AM fungi. Trends Plant Sci..

[29] Kohlen W (2012). The tomato carotenoid cleavage dioxygenase8 (SlCCD8) regulates rhizosphere signaling, plant architecture and affects reproductive development through strigolactone biosynthesis. New Phytol..

[30] Al-Babili S, Bouwmeester HJ (2015). Strigolactones, a novel carotenoid-derived plant hormone. Annu. Rev. Plant Biol..

[31] Booker J (2004). MAX3/CCD7 is a carotenoid cleavage dioxygenase required for the synthesis of a novel plant signaling molecule. Curr. Biol..

[32] Booker J (2005). MAX1 encodes a cytochrome P450 family member that acts downstream of MAX3/4 to produce a carotenoid-derived branch-inhibiting hormone. Dev. Cell.

[33] Das M (2015). Parasitic plants Striga and Phelipanche dependent upon exogenous strigolactones for germination have retained genes for strigolactone biosynthesis. Am. J. Plant Sci..

[34] Hacham Y, Hershenhorn J, Dor E, Amir R (2016). Primary metabolic profiling of Egyptian broomrape (*Phelipanche aegyptiaca*) compared to its host tomato roots. J. Plant Physiol..

[35] Schaffer A, Jacobsohn R, Joel D, Eliassi E, Fogelman M (1991). Effect of broomrape (Orobanche spp.) infection on sugar content of carrot roots. Hortscience.

[36] Alcántara E, Morales-García M, Díaz-Sánchez J (2006). Effects of broomrape parasitism on sunflower plants: growth, development, and mineral nutrition. J. Plant Nutr..

[37] Labrousse P, Arnaud M, Serieys H, Bervillé A, Thalouarn P (2001). Several mechanisms are involved in resistance of Helianthus to *Orobanche cumana* Wallr. Ann. Bot..

[38] Aly R (2009). Gene silencing of mannose 6‐phosphate reductase in the parasitic weed *Orobanche aegyptiaca* through the production of homologous dsRNA sequences in the host plant. Plant Biotechnol. J..

[39] Westwood, J. H. In *Parasitic Orobanchaceae* (eds Joel, D. M., Gressel, J. & Musselman, L. J.) Ch. 6, 87–114 (Springer, 2013).

[40] Nandula VK, Foster JG, Foy CL (2000). Impact of *Egyptian broomrape* (*Orobanche aegyptiaca* (Pers.) parasitism on amino acid composition of carrot (*Daucus carota* L.). J. Agr. Food Chem..

[41] Perrin F (2017). Combined *Alternaria dauci* infection and water stresses impact carotenoid content of carrot leaves and roots. Environ. Exp. Bot..

[42] Kidmose U., Hansen S. L., Christensen L. P., Edelenbos M., Larsen E., Nørbaek R. (2006). Effects of Genotype, Root Size, Storage, and Processing on Bioactive Compounds in Organically Grown Carrots (Daucus carota L.). Journal of Food Science.

[43] Clermont K (2019). Comparative metabolomics of early development of the parasitic plants P*helipanche aegyptiaca* and *Triphysaria versicolor*. Metabolites.

[44] Delavault P (2002). Isolation of mannose 6‐phosphate reductase cDNA, changes in enzyme activity and mannitol content in broomrape (*Orobanche ramosa*) parasitic on tomato roots. Physiologia Plantarum.

[45] Pageau K, Simier P, Le Bizec B, Robins RJ, Fer A (2003). Characterization of nitrogen relationships between Sorghum bicolor and the root‐hemiparasitic angiosperm *Striga hermonthica* (Del.) Benth. using K15NO3 as isotopic tracer. J. Exp. Bot..

[46] Gaudin Z (2014). Robust method for investigating nitrogen metabolism of ^15^N labeled amino acids using AccQ. Tag ultra performance liquid chromatography-photodiode array-electrospray ionization-mass spectrometry: application to a parasitic plant–plant interaction. Anal. Chem..

[47] Abbes Z, Kharrat M, Delavault P, Chaïbi W, Simier P (2009). Nitrogen and carbon relationships between the parasitic weed O*robanche foetida* and susceptible and tolerant faba bean lines. Plant Physiol. Biochem..

[48] Singh M, Singh D, Misra P, Tewari K, Krishnan P (1968). Biochemical aspects of parasitism by the angiosperm parasites: starch accumulation. Physiol. Plant..

[49] Dawson JH, Musselman LJ, Wolswinkel P, Dörr I (1994). Biology and control of Cuscuta. Reviews of Weed Science.

[50] Aber M, Fer A, Sallé G (1983). Etude du transfert des substances organiques de l’hôte (*Vicia faba*) vers le parasite *(Orobanche crenata* Forsk.): Transfer of Organic substances from the host plant *Vicia faba* to the parasite *Orobanche crenata* Forsk. Z. Pflanzenphysiol.

[51] Tomilov AA, Tomilova NB, Wroblewski T, Michelmore R, Yoder JI (2008). Trans‐specific gene silencing between host and parasitic plants. Plant J..

[52] Nandula, V. K., Foy, C. L. & Orcutt, D. M. Glyphosate for *Orobanche aegyptiaca* control in *Vicia sativa* and *Brassica napus*. *Weed Sci*., 486–491 (1999).

[53] Roney JK, Khatibi PA, Westwood JH (2007). Cross-species translocation of mRNA from host plants into the parasitic plant dodder. Plant Physiol..

[54] Gal-On A (2009). Broomrape can acquire viruses from its hosts. Phytopathology.

[55] Hamamouch N (2005). A peptide from insects protects transgenic tobacco from a parasitic weed. Transgenic Res..

[56] Liu Q (2014). Striga hermonthica MAX2 restores branching but not the very low fluence response in the *Arabidopsis thaliana* max2 mutant. New Phytol..

[57] Ibdah M (2006). Functional characterization of CmCCD1, a carotenoid cleavage dioxygenase from melon. Phytochemistry.

