# Supplementary material for: Broomrape infestation in carrot (Daucus carota): Changes in carotenoid gene expression and carotenoid accumulation in the parasitic weed Phelipanche aegyptiaca and its host
Source: Sci Rep. 2020 Jan 15;10:324. doi: 10.1038/s41598-019-57298-7 (PMC6962276; doi:10.1038/s41598-019-57298-7)
Supplement: Supplementary file 1 — Supplementary Table [file 41598_2019_57298_MOESM1_ESM.docx]

| Primer description | Sequence (5' - 3') |
| --- | --- |
| *DcPSY1* | Forward-5'- GGG CAA TCT ATG TGT GGT GTA G -3'  Reverse-5'- TTC AGC CTC TTC TCC CAT CT -3' |
| *DcCRTISO* | Forward-5'- TGT GAT TGG GTC TGG GAT TG -3'  Reverse-5'- GCT CCC ACC AGG AAT CAA ATA -3' |
| *DcD27* | Forward-5'- CTA TCG CGG AGT GTA CAA GAA A-3'  Reverse-5'- CTA GTT GTT GGT CTG GGC TAA A -3' |
| *DcCCD7* | Forward-5'- AAG CCA AAC CCA GCC TAA A -3'  Reverse-5'- CGG AAC GTT GGG AGA TGA ATA A -3' |
| *DcCCD8* | Forward-5'- GCG TAT GAG CAA ACA GAT GAA -3'  Reverse-5'- TGA GCT TAT CTA GAA TTG TGG TGT -3' |
| *DcActin* | Forward-5'- AAG AGC ATC TGC CAC TAC AC -3'  Reverse-5'- GGC AGC TCG TAG TTC TTC TC -3' |
| *PaD27* | Forward-5'- CGA TCC CTA GAT CCA GCT TT -3'  Reverse-5'- CTG CAT TTC TGT TGG TGT CTT -3' |
| *PaCCD7* | Forward-5'-CAC AGC CAA CGT ACA TAC TCT C -3'  Reverse-5'- GAT GGT GGT GGT GTC TTG TT -3' |
| *PaCCD8* | Forward-5'- CGT CAC GGA TAA CGA GTT TCT -3'  Reverse-5'- CAC CTT CCT CTC GTT CGT TC-3' |
| *PaActin* | Forward-5'- AAT GAT CGG AAT GGA AGC TG -3'  Reverse-5'- TCC ACT GAA GGA CGA TGT TTC -3' |

**Supplemental Table S1.** Synthetic oligonucleotides used for qRT-PCR expression of carrot (*D. carota*) and *P. aegyptiaca* *PSY1*, *CRTISO*, *D27*, *CCD7,* *CCD8* and the housekeeping gene actin in roots of the different carrot cultivars and in the tubercles of the parasitic organs.
