# Supplementary figures and images for: Broomrape infestation in carrot (Daucus carota): Changes in carotenoid gene expression and carotenoid accumulation in the parasitic weed Phelipanche aegyptiaca and its host
Source: Sci Rep. 2020 Jan 15;10:324. doi: 10.1038/s41598-019-57298-7 (PMC6962276; doi:10.1038/s41598-019-57298-7)

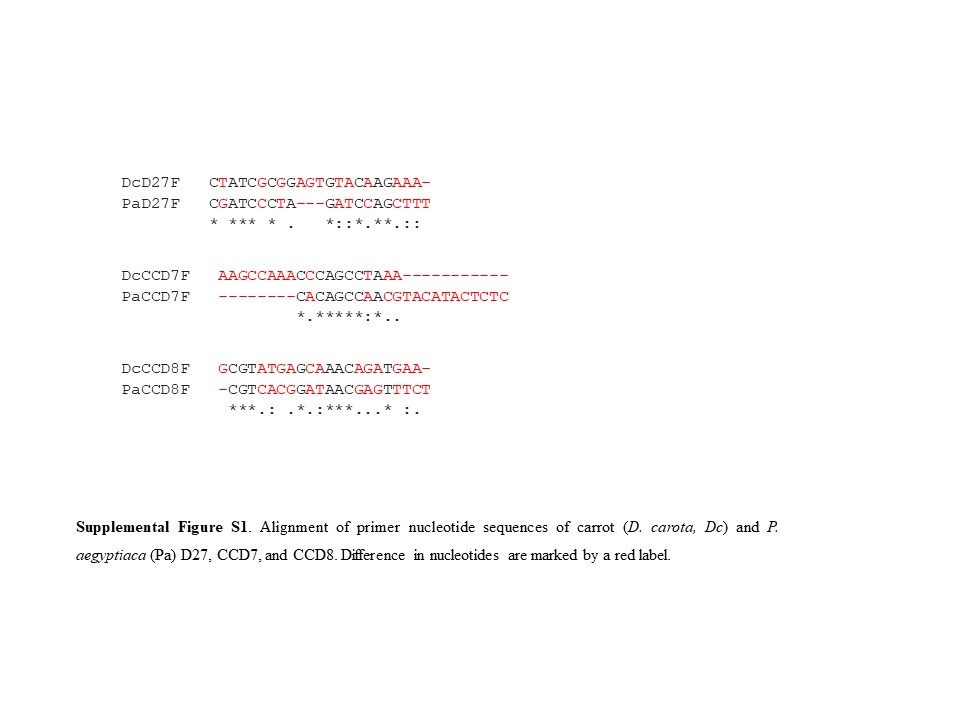

Supplement: Supplementary file 2 — Supplementary Information [file 41598_2019_57298_MOESM2_ESM.jpg]
